# The re-emergence of sexually transmissible multidrug resistant *Shigella flexneri* 3a, England, United Kingdom

**DOI:** 10.1038/s44259-024-00038-3

**Published:** 2024-08-02

**Authors:** Lewis C. E. Mason, Hannah Charles, Katie Thorley, Charlotte E. Chong, P. Malaka De Silva, Claire Jenkins, Kate S. Baker

**Affiliations:** 1 NIHR HPRU in Gastrointestinal Infections at University of Liverpool, Liverpool, UK; 2 Department of Clinical Infection, Microbiology, and Immunology; Institute for Infection, Veterinary and Ecological Sciences, University of Liverpool, Liverpool, UK; 3 UK Health Security Agency (UKHSA), London, UK; 4 Department of Genetics, University of Cambridge, Cambridge, CB2 3EH UK

**Keywords:** Bacterial genes, Bacterial infection, Gastroenteritis, Antimicrobial resistance, Computational biology and bioinformatics

## Abstract

Shigellosis is an enteric infection that transmits through the faecal-oral route, which can occur during sex between men who have sex with men (MSM). Between 2009 and 2014, an epidemic of sexually transmissible *Shigella flexneri* 3a occurred in England that subsequently declined. However, from 2019 to 2021, despite SARS-CoV-2 restrictions, *S. flexneri* 3a continued to re-emerge. We explored possible drivers of re-emergence by comparing host demography and pathogen genomics. Cases were primarily among 35–64 year old men in London. Genomic analyses of 502 bacterial isolates showed that the majority (58%) of re-emerging MSM strains were a clonal replacement of the original, with reduced antimicrobial resistance, conservation of plasmid col156_1, and two SNPs with 19 predicted effects. The absence of major changes in the pathogen or host demographics suggest that other factors may have driven the re-emergence of *S. flexneri* 3a and highlight the need for further work in the area.

## Introduction

Bacteria in the *Shigella* genus are Gram-negative, non-motile, and highly pathogenic to humans. There are four species of *Shigella*: *boydii, dysenteriae, flexneri* and *sonnei*. As few as 10–100 colony forming units of *Shigella* bacteria can cause shigellosis^1^, a highly infectious enteric illness^2^. The low infectious dose, in combination with potential asymptomatic carriage, allows *Shigella* to propagate unbeknownst between hosts^3^. Symptoms of shigellosis are fever, nausea, bloody diarrhoea, and stomach cramping^2^. Rarer and more severe complications of gastrointestinal shigellosis include haemolytic uremic syndrome^4^, toxic megacolon^5^, bacteraemia^6^, and reactive arthritis^7^. *Shigella* spp. are transmitted through the faecal-oral route, most commonly associated with person to person transmission. On a global basis, transmission is common in institutional^8^, and community settings^9^, and *Shigella* spp. are also transmitted through consumption of contaminated food and water^10^, and is a major cause of travel-associated illness in higher-income nations.

In higher-income nations, since the 1970s^11–13^, shigellosis transmission has also been described as a sexually transmissible illness among men who have sex with men (MSM). The first UK outbreak of MSM-associated shigellosis transmission was identified in 2004^14^. Since then, MSM-associated outbreaks have continued to increase in frequency in the UK, and other nations^15^. This is associated with engagement in higher-risk sexual activities, such as chemsex (the use of specific drugs prior to or during sexual activity, as an enhancer or facilitator)^16^, anal-digital contact, and condomless anal intercourse^16^. In line with global trends, sexually transmissible shigellosis among MSM is most commonly associated with *Shigella flexneri* (*S. flexneri*) and *Shigella sonnei* (*S. sonnei*)^17^.

Antimicrobial resistant (AMR) *Shigella* spp. have been described since the 1960s, when resistance to streptomycin, sulphonamides, tetracycline, and ampicillin became common^18,19^. Subsequently, resistance to ciprofloxacin^20^, and azithromycin^21^, were identified in *S. sonnei* and *S. flexneri*. AMR *Shigella* spp. are a growing cause for concern, particularly in sexual transmission networks, as multiple drug resistant and extensively drug resistant (XDR) strains of *Shigella* spp. are on the rise^22^, with the *bla*_CTX-M_ family of genes - conferring ceftriaxone resistance - being described across *Shigella* spp.^23^, and recently causing an international outbreak of XDR *S. sonnei*^22^. These resistance profiles are especially concerning, as The World Health Organization (WHO) currently recommends ciprofloxacin to be used as the first line of treatment, followed by azithromycin and ceftriaxone as second line treatments (alongside pivmecillinam)^24^.

In 2009 in the UK, transmission of *Shigella* spp. associated with domestically acquired infection in adult males, a proxy for MSM-associated sexual transmission, became increasingly common and was originally associated with increases in *S. flexneri* serotype 3a. Associated drivers were subsequently identified as sexual encounters through social media and smartphone applications, and engaging in higher-risk behaviours, such as chemsex, and condomless sexual intercourse^16^. Following the 2009 epidemic of *S. flexneri* 3a, there were overlapping *S. flexneri* 2a and *S. sonnei* outbreaks^25^, driven by the acquisition of a plasmid encoding resistance to azithromycin^21^. Since 2015, domestic acquisition of shigellosis among MSM in the UK has been dominated by ciprofloxacin resistant *S. sonnei*, thought to have been imported from Asia and containing triple mutations in the quinolone resistance-determining region (QRDR)^26^. This *S. sonnei* lineage has latterly (2021 onwards) become XDR through acquisition of an extended-spectrum beta lactamase (ESBL) encoding plasmid^22,27^, that has also transferred into *S. flexneri* 2a^28^. The spread of AMR shigellosis may be exacerbated by behavioural changes associated with the use of pre-exposure prophylaxis (PrEP) for human immunodeficiency virus^3,27^.

Between 2015 and 2018, the number of *S. flexneri* 3a cases fell to below pre-epidemic (2004–2008) levels. However, *S. flexneri* 3a increased again in the UK from 2019 to 2021, despite the non-pharmaceutical interventions associated with the SARS-CoV-2 pandemic that brought about a general reduction in gastrointestinal disease^29^. Here, we explored whether changes in host demographic profiles or pathogen evolution played a role in the re-emergence of sexually transmissible *S. flexneri* 3a. Understanding the drivers of the emergence of new subtypes of sexually transmissible shigellosis is key to intervening in the worsening AMR profile and likely increased transmission scenario. The aim of our study was to integrate whole genome sequencing (WGS) data with enhanced epidemiological data to explore how interactions between bacteria, host, and environmental factors may have initially driven the re-emergence of *S. flexneri* 3a in England, which continued despite the impact of the SARS-CoV-2 pandemic on pathogen transmission.

## Results

### Overview of demographic data

**Fig. 1:**
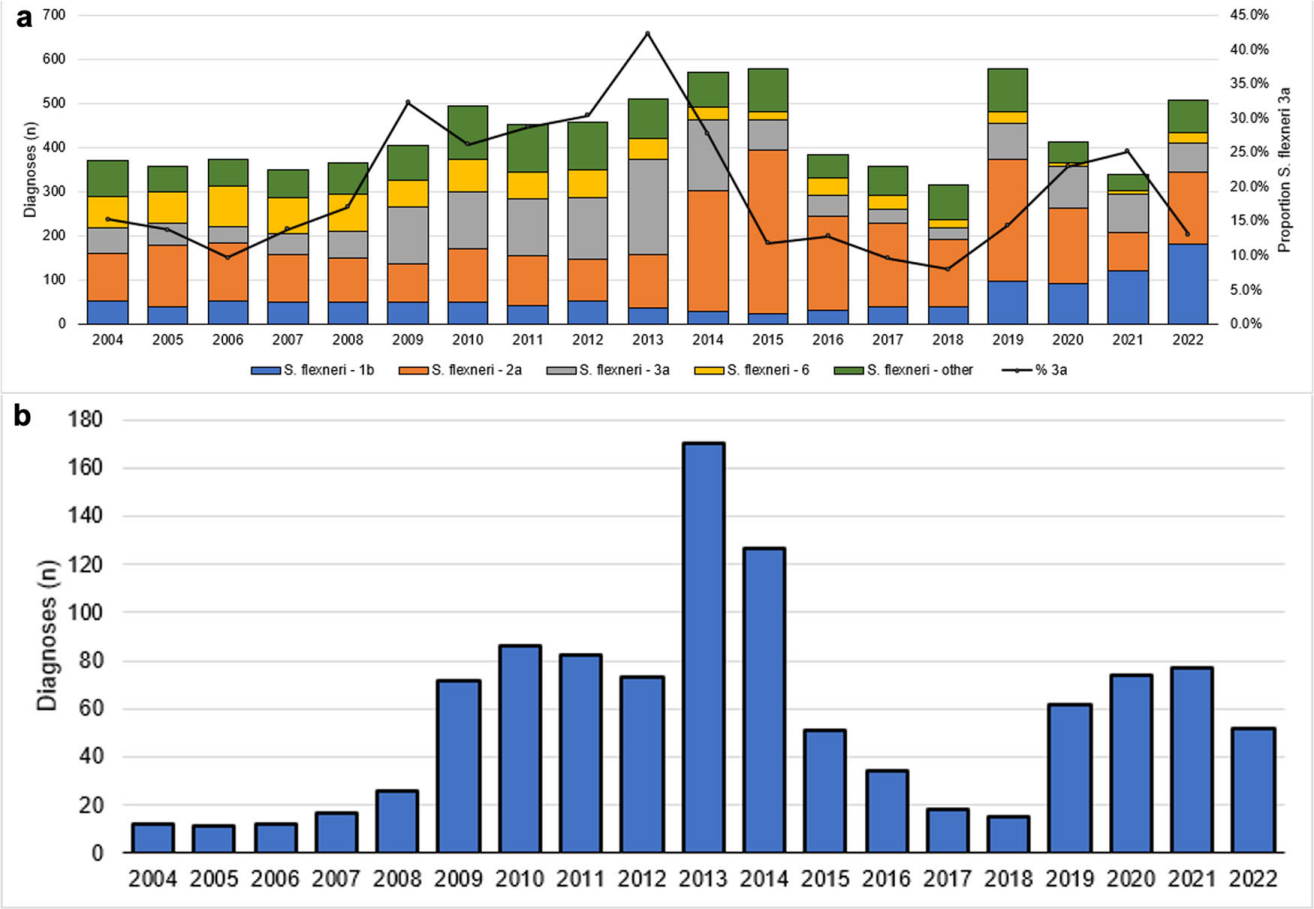
*Shigella flexneri* 3a diagnoses in the UK in context. **a** Number of *S. flexneri* diagnoses by serotype and proportion *S. flexneri* 3a, England, 2004 to 2022. **b** Number of *S. flexneri* 3a diagnoses between 2004 and 2022 among presumptive men who have sex with men (MSM), data obtained from the Gastro Data Warehouse (GDW).

Between 2004 and 2020, 40.5% (*n* = 6864/16,952) of *Shigella* spp. diagnoses among adults processed by the Gastrointestinal Bacteria Reference Unit (GBRU) at the UKHSA were identified as *S. flexneri*. Shigellosis is a notifiable disease in the UK, with mandatory isolate referral for *S. flexneri*, highlighting the representativeness of our data. Following an increase in the proportion of *S. flexneri* cases that were serotype 3a between 2004 and 2013, and a decline between 2013 and 2018, the proportion of 3a more than tripled between 2018 and 2020 (from 7.8% [*n* = 7/90] to 30.0% [*n* = 85/287], Fig. 1a). Epidemiological comparisons of *S. flexneri* 3a cases among presumptive MSM in the epidemiological emergence phase (2012–2013) and epidemiological re-emergence phase (2019–2020) (Table 1), showed that the age and geographical distribution remained largely unchanged, with the greatest proportion of cases reported from London and the Southeast of England (Table 1). Hospitalisation data was not available for the cases identified during the emergence phase; however, enhanced surveillance questionnaires from the re-emergence phase indicate that 35% (*n* = 18/51) of presumptive MSM were hospitalised. WGS has revealed that the re-emergence of *S. flexneri* 3a has been driven mainly by a single 10 SNP cluster t10.1189, (*n* = 108/128, 84.4%) (Table 1).

**Table 1:** Summary of characteristics of the recent re-emergence of *S. flexneri* 3a cases among presumptive MSM and comparison to original emergence, England

Characteristic	2012–2013 *n* (%)	2019–2020 *n* (%)
Age		
16–34	95 (39.7)	54 (42.2)
35–64	136 (56.9)	67 (52.3)
65+	5 (2.1)	7 (5.5)
Unknown	3 (1.3)	0 (0.0)
Median age [IQR]^a^	38.5 [30–46.5]	36 [29–45.5]
Region		
London	140 (58.6)	91 (71.1)
South East	36 (15.1)	20 (15.6)
North West	28 (11.7)	2 (1.6)
West Midlands	8 (3.4)	1 (0.8)
East of England	13 (5.4)	8 (6.3)
South West	6 (2.5)	4 (3.1)
Yorkshire & Humber	2 (0.8)	1 (0.8)
North East	4 (1.7)	1 (0.8)
East Midlands	2 (0.8)	0 (0.0)
Unknown	0 (0.0)	0 (0.0)
Hospitalised		
Yes	ND	18 (35.3)
No	ND	33 (64.7)
Median nights in hospital [IQR]^a^		2 [2–4]
10 SNP^**b**^ cluster		
t10.1177	27 (11.3)	0 (0.0)
t10.43	18 (7.5)	0 (0.0)
t10.138	2 (0.8)	0 (0.0)
t10.116	1 (0.4)	0 (0.0)
t10.1182	1 (0.4)	0 (0.0)
t10.1197	1 (0.4)	0 (0.0)
t10.1211	1 (0.4)	0 (0.0)
t10.1227	1 (0.4)	0 (0.0)
t10.1356	1 (0.4)	0 (0.0)
t10.1189	0 (0.0)	108 (84.4)
t10.1305	0 (0.0)	2 (1.6)
t10.1627	0 (0.0)	1 (0.8)
t10.1648	0 (0.0)	1 (0.8)
t10.1684	0 (0.0)	13 (10.2)
t10.1852	0 (0.0)	1 (0.8)
t10.1969	0 (0.0)	1 (0.8)
Unknown	186 (77.8)	1 (0.8)

^a^Interquartile range (IQR).

^b^Single nucleotide polymorphism (SNP).

### *S. flexneri* 3a re-emergence featured clonal replacement

**Fig. 2:**
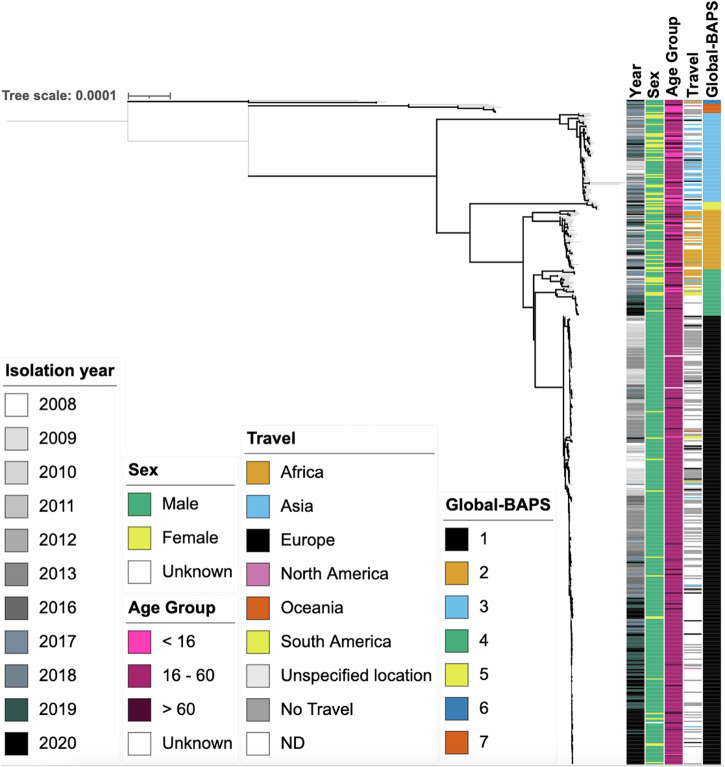
A midpoint rooted maximum likelihood phylogenetic tree showing the associations between UK isolates of *Shigella flexneri* 3a and travel (*n* = 502). Metadata tracks show, year, sex, age group, travel, and BAPS subgroup, coloured according to the inlaid keys. Emboldened branches represent a bootstrap value of ≥70 out of 100. Branches represent substitutions across a 22,985 bp alignment. The reference genome used was 4,522,047 bp in length.

To determine the genomic epidemiology among 502 isolates of *S. flexneri* 3a from the bacterial clade emergent and re-emergent periods (2008–2013 *n* = 205; 2016–2020 *n* = 298), a phylogenetic tree was constructed and matched to epidemiological metadata (Fig. 2). This was complemented by Bayesian Analysis of Population Structure (BAPS) groups being computed to assign genetically similar clusters, which we called Global-BAPS clusters. This revealed that isolates clustered by patient travel destination, with isolates from cases reporting recent travel to Asia mostly (71%, *n* = 25/35) belonging to the Global-BAPS-3 cluster, while African-travel associated isolates typically (74%, *n* = 26/35) belonged to Global-BAPS-2 (Table 2, Fig. 2). Notably, more than half (51%, *n* = 257/502) of the isolates have no associated travel data. Global-BAPS-1 cluster was mostly (84%, *n* = 286/339) composed of aged 16–60 males, who were without a known history of international travel, and was thus defined as MSM-associated. The majority (78%, *n* = 159/205) of the 2008–2014 *S. flexneri* 3a outbreak belonged to the Global-BAPS-1 cluster, as did the majority (58%, *n* = 180/312) of *S. flexneri* 3a isolated between 2016 and 2020.

**Table 2:** Temporal and demographic features of isolates in this study by Global-BAPS groups

		Year (%)											Sex (%)		Age group in years (%)			Travel (%)						
Global-BAPS Group	Total (*n*)	2008	2009	2010	2011	2012	2013	2016	2017	2018	2019	2020	Male	Female	<16	16–60	>60	Africa	Asia	Europe	N-America	Oceania	S-America	No Travel Reported
1	339	4	4	8	6	5	19	10	5	2	14	22	96	4	1	94	5	0	1	4	0.3	0.3	1	93.5
2	45	2	16	0	2	2	11	9	16	7	22	13	76	24	11	69	20	58	2	0	0	0	0	37.7
3	67	3	7	6	9	4	4	16	15	16	15	3	64	36	24	63	13	0	37	6	0	0	0	55.2
4	35	3	3	3	6	3	3	9	11	14	23	23	71	29	9	80	11	23	3	0	0	0	11	62.8
5	6	0	0	17	0	0	0	0	0	17	50	17	33	67	17	67	17	0	67	0	0	0	0	16.6
6	3	0	0	0	0	0	0	0	0	67	0	33	100	0	0	100	0	33	0	0	0	0	0	66.7
7	7	0	0	0	0	0	0	14	43	14	29	0	71	29	57	29	14	0	0	0	14	0	0	85.7

**Fig. 3:**
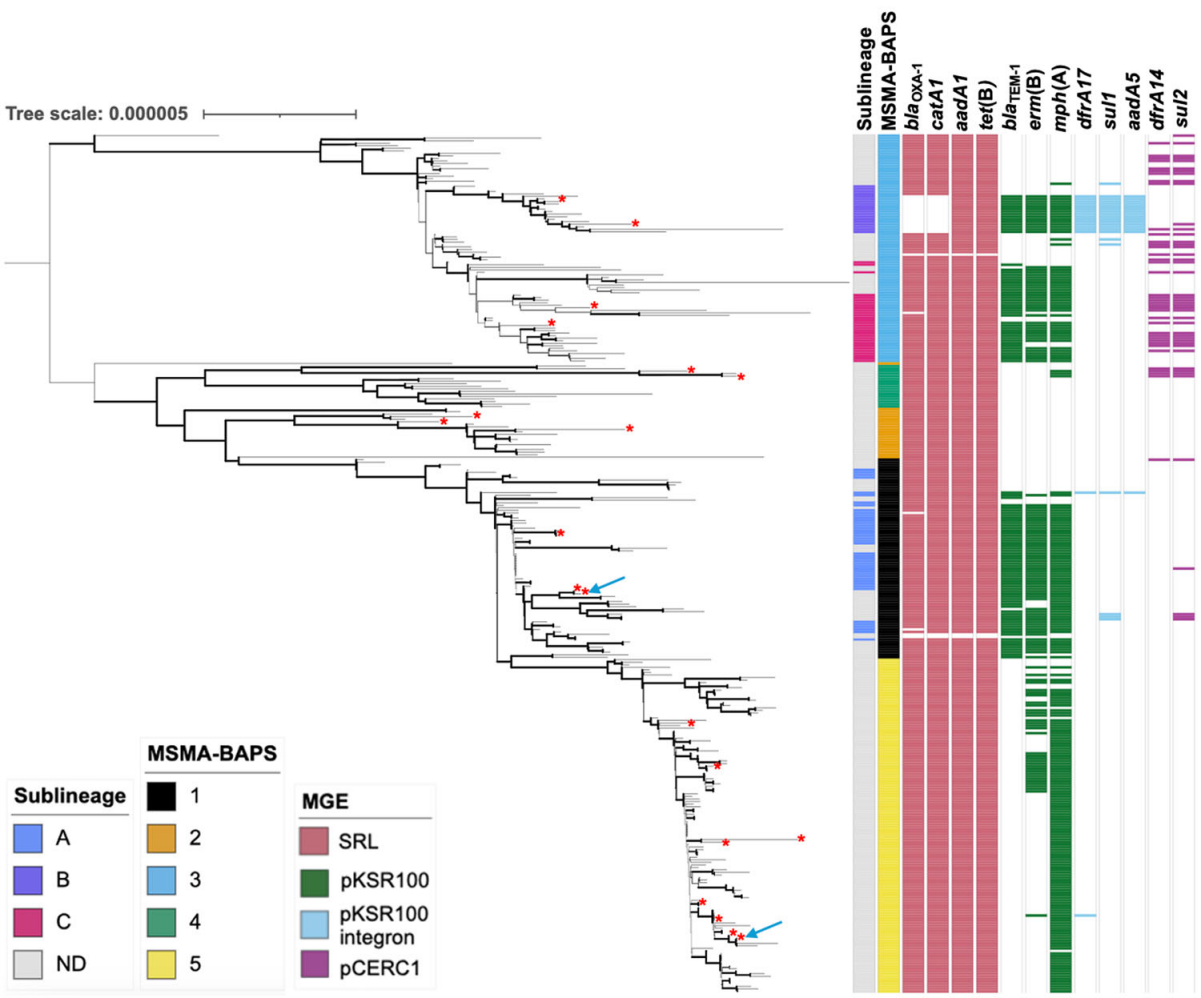
Demography, genotypic, and AMR features of Global-BAPS-1 isolates. A midpoint rooted maximum likelihood phylogenetic tree features of Global-BAPS-1 *Shigella flexneri* 3a (*n* = 339). Metadata tracks show genomic subtypes: previously designated sublineage (from ref. ^30^) and MSMA-BAPS subtype, followed by the presence of selected AMR genes. Genes are grouped and coloured according to their commonly associated mobile genetic element: *Shigella* resistance locus (pink), pKSR100 (green), pKSR100 integron (blue), pCERC1 (purple). Emboldened branches represent a bootstrap value of ≥70 out of 100. Red asterisks at the end of branches represent the isolate being used in minimum inhibitory concentration (MIC) experiments (*n* = 20). A blue arrow represents Nanopore sequencing for plasmid analysis being performed on an isolate (*n* = 2). Branches represent expected number of substitutions per site across a 1560 bp alignment. The reference genome used was 4,522,047 bp in length.

As we were focused on pathogen changes between the domestically transmitted emergent and re-emergent Global-BAPS-1 clone which form the majority 68% (*n* = 339/502) of *S. flexneri* 3a, we conducted a further round of BAPS grouping to facilitate study at a finer resolution. These more highly resolved groups are referred to as MSMA-BAPS groups (Table 3, Fig. 3). Correlation with the nomenclature from the previous study of the emergent event revealed that lineages, previously designated as sublineages B and C^30^, were associated with MSMA-BAPS-1, and sublineage A with MSMA-BAPS-3, while cases from 2019 onwards were now MSMA-BAPS-5. This revealed that the first outbreak was majorly associated with MSMA-BAPS-1 (23%) and BAPS-3 (39%), and the second outbreak was associated with MSMA-BAPS-5 (42%), (Table 3). Thus, this subsequent analysis revealed further distinction between the two time periods, where the recent *Shigella* re-emergence (2016–2020) was associated with a single clonal expansion that replaced the earlier outbreak (Fig. 3). Thus, MSMA-BAPS-1 (originally reported as MSM-associated clade^30^) has been clonally replaced by MSMA-BAPS-5. Pairwise single nucleotide polymorphism (SNP) analyses further supported the distinction of the two groups with distances between isolates in MSMA-BAPS-1 and MSMA-BAPS-5 (Mean: 36, Range: 20 – 76) being greater than within either MSMA-BAPS-1 (Mean: 19, Range: 0–63) or MSMA-BAPS-5 (Mean: 14, Range: 0–41), consistent with previously observed evolution in this lineage^30^, (Supplementary Table 1).

**Table 3:** Temporal features of the Global-BAPS-1 cluster broken down into MSMA-BAPS groups

		Year (%)										
MSMA- BAPS Group	Total (*n*)	2008	2009	2010	2011	2012	2013	2016	2017	2018	2019	2020
1	79	3	1	3	4	8	42	27	8	4	1	1
2	21	24	33	38	0	0	0	0	5	0	0	0
3	90	2	4	17	18	12	36	8	2	0	1	0
4	17	29	12	12	12	6	0	18	6	0	0	6
5	132	0	0	0	0	0	0	2	5	3	35	56

### Re-emergent *S. flexneri* 3a feature non-AMR genetic changes

To explore accessory genome changes in the clonal replacement of MSMA-BAPS-1 by MSMA-BAPS-5, we inspected the plasmid content of the isolates. This revealed the conservation of a 16,667 bp length col156_1 plasmid in MSMA-BAPS-5 (100%, *n* = 132/132) compared to MSMA-BAPS-1 (5%, *n* = 4/79), (Supplementary Table 2). The plasmid sequence was 99.74% identical across 99% length with an *E. coli* plasmid (GenBank accession CP133872.1) and was predicted to contain multiple copies of genes associated with mobilisation, replication, LysE translocation and methyltransferase. Owing to the recently described importance of colicins in *Shigella* spp.^31^, we also explored the composition of colicin encoding genes across Global-BAPS-1. This revealed no colicin-related differences between MSMA-BAPS-1 and MSMA-BAPS-5, but did show that two colicin genes (encoding tolR and colicin I receptor) were highly conserved as a pair (99.5%, *n* = 210/211) in Global-BAPS-1 (Supplementary Table 3). We investigated the possibility of changes in the critical virulence factor, the *Shigella* invasion plasmid, but found no discernible difference in the mapping coverage between MSMA-BAPS 1 and MSMA-BAPS-5 isolates (Supplementary Fig. 2), or in virulence gene content (Supplementary Data 1). Finally, we identified two SNPs on the ancestral node beginning the MSMA-BAPS-5 cluster in comparison to the ancestral node ending the MSMA-BAPS-1 cluster. These two SNPs had a total of 19 predicted gene effects: upstream (68%, *n* = 13/19), downstream (26%, *n* = 5/19), and missense (5%, *n* = 1/19) (Supplementary Table 4), though the upstream and downstream effects (defined as where the SNP is within 5000 bases of a gene) were designated as ‘modifiers’, where *‘predictions are difficult or there is no evidence of impact*.’^32^ The single missense effect resulted in an amino acid substitution of alanine (A) with threonine (T) in the lptE protein associated with lipopolysaccharide in the outer membrane (Supplementary Table 5). The BLOcks SUbstitution Matrix 62 (BLOSUM) assigned this substitution a score of ‘zero’, meaning the replacement is likely to be conservative and have little impact on protein function.

### Re-emergent *S. flexneri* 3a is less antimicrobial resistant

Owing to the importance of shifting resistance profiles in previous MSM-associated shigellosis outbreaks, we explored whether there were differences in the emergent and re-emergent subtypes, with respect to AMR. We identified the antimicrobial resistance genes (ARGs) across the isolates and correlated this with genomic groupings (Fig. 3, Table 4). This revealed that emergent MSMA-BAPS-1 isolates had highly conserved *erm*(B) (73%, *n* = 58/79), *mph*(A) (77%, *n* = 61/79) and *bla*_TEM-1_ (80% *n* = 63/79) genes, whereas a loss of, first, *bla*_TEM-1_ and then *erm*(B) ARGs in the re-emergent MSMA-BAPS-5 isolates was observed. Although *bla*_TEM-1_ and then *erm*(B) genes were lost, *mph*(A) has been maintained in MSMA-BAPS-5 isolates (92%, *n* = 121/132). Previous work on this lineage revealed that most ARGs were carried on four main mobile genetic elements (MGEs); the *Shigella* resistance locus *bla*_OXA-1_, *catA1, aadA1*, and *tet(B)*, pKSR100 (*bla*_TEM-1_, *erm*(B), and *mph*(A)), pKSR100 integron (*dfrA17*, *sul1 and aadA5)* or pCERC1 (*dfrA14 and sul2)*^30^. Grouping ARGs by these MGEs suggested that, in contrast to the emergent event associated with MSMA-BAPS-4 where 24% (*n* = 4/17) of isolates contain *dfrA14* and *sul2, indicative of pCERC1 presence*, only a single MSMA-BAPS-5 isolate potentially contains the pKSR100 integron (indicated by presence of *dfrA17, though absence of sul1* and *aadA5*, 0.75%, *n* = 1/133), and none contain genes associated with pCERC1 (indicated by genes *dfrA14* and *sul2*, 0%, *n* = 0/133).

**Table 4:** Proportion of *S. flexneri* 3a MSMA-BAPS isolates containing various ARGs which demonstrated change between clusters

	Proportion of MSMA-BAPS cluster containing gene (%)					
Antimicrobial class	Gene	1 (*n* = 79)	2 (*n* = 21)	3 (*n* = 90)	4 (*n* = 17)	5 (*n* = 132)
Aminoglycosides	***aph(3”)-lb***	4	0	37	24	0
	***aadA5***	3	0	17	0	0
	***aph(6)-ld***	4	0	39	24	0
Beta-lactams	***bla***_**TEM-1**_	80	0	57	0	0
Trimethoprim	***dfrA14***	3	0	38	24	0
	***dfrA17***	3	0	17	0	1
Macrolides	***erm*(B)**	73	0	53	0	26
	***mph*(A)**	77	0	57	18	92
Sulphonamides	***sul1***	6	0	20	0	0
	***sul2***	8	0	40	24	0
Antiseptics	***qacE***	1	0	17	0	0

Phenotypic testing (minimum inhibitory concentrations [MICs]) of the antimicrobials corresponding to the genotypic resistance profiles was performed to investigate whether changes in the genotype of the bacteria influenced phenotypic antimicrobial resistance (AMR). The findings largely corresponded (90%, *n* = 18/20 concordance) with genotypic resistance (Supplementary Table 6). Specifically, the presence of at least one of *erm*(B) or *mph*(A) and the isolate being resistant to azithromycin had a 95% (*n* = 19/20) concordance. Presence of at least one pair of a *sul* and *dfr* gene, and the isolate being resistant to trimethoprim-sulfamethoxazole had a 90% (*n* = 18/20) concordance. This revealed that Global-BAPS-1 *S. flexneri* 3a in the UK has acquired resistance to azithromycin and trimethoprim-sulfamethoxazole, primarily through acquisition of *mph*(A), and *sul1* and *sul2*. None of the isolates were resistant to ciprofloxacin, consistent with there being no relevant QRDR mutations (i.e., ParC S80I, nor GyrA S83L or D87G) present. Also, none of the isolates were resistant to ceftriaxone. While resistance to azithromycin correlated with the presence of either *erm*(B) or *mph*(A), higher levels of resistance (>256 mg/L) were significantly associated with the presence of both genes (85% [*n* = 17/20] concordance, chi-square test value 10.444; *p* = 0.0012) (Supplementary Table 6). All isolates were sensitive to fosfomycin or mecillinam.

### AMR gene loss on pKSR100 is facilitated by transposases

**Fig. 4:**
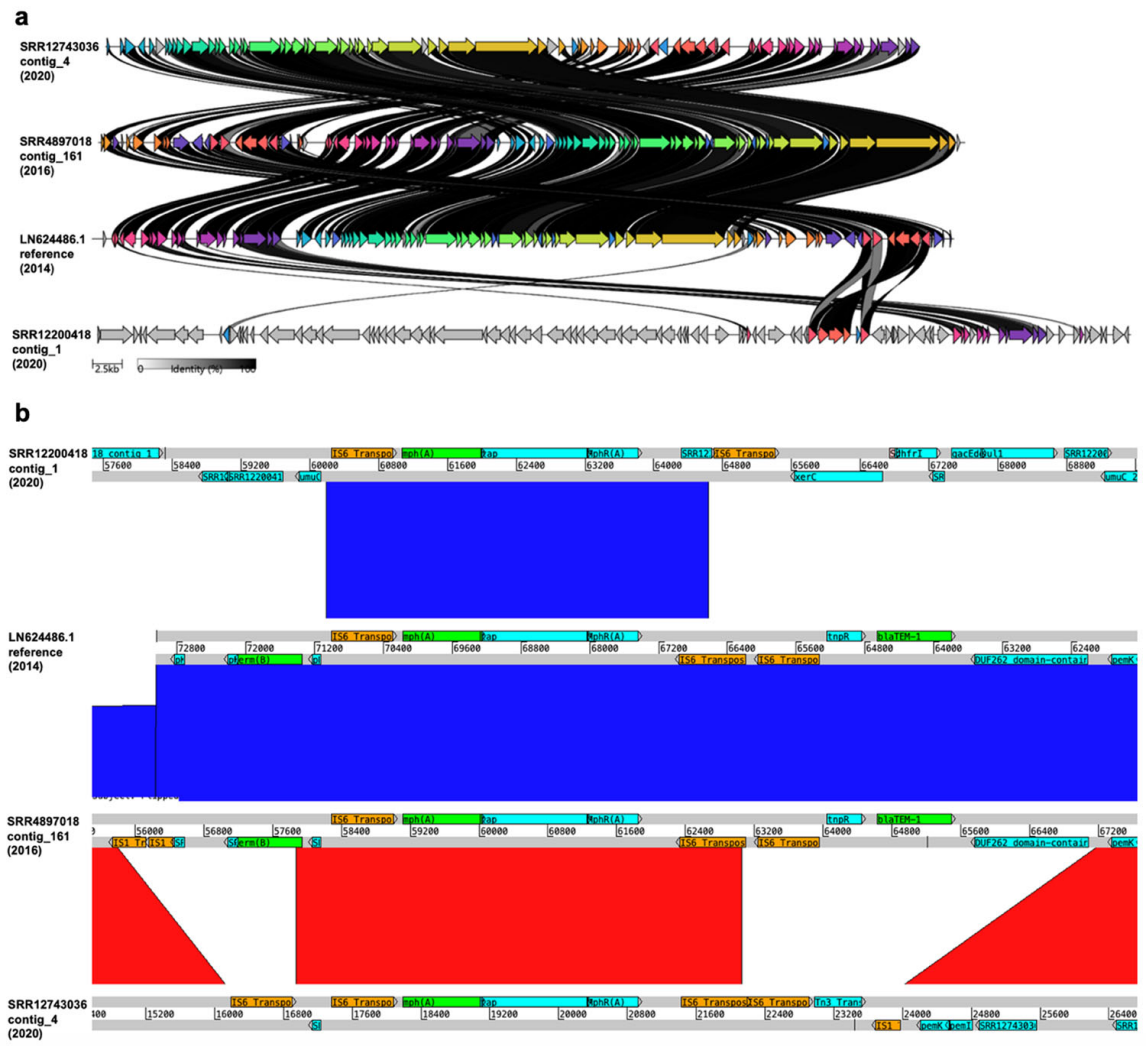
Comparison of plasmids found in *S*. *flexneri* 3a. **a** A diagram showing an overall comparison of four plasmids from *S. flexneri* 3a. Coloured arrows represent a similarity of ≥30% between two plasmids, while grey arrows represent a similarity of <30%. The shade of the lines between specific genes represents varying % identity. **b** A comparison of four plasmids from *S. flexneri* 3a. Red bands represent forward matches, while blue bands represent reverse matches. Genes of interest were annotated manually, by corroborating results between NCBI-AMR-Finder-Plus, Prokka annotation, and BLASTp. Genes highlighted in green are the three main AMR genes of interest. Genes highlighted in orange are in the IS1 and IS6 transposase families. All comparison windows were set to only show shared sequences of at least 999 bp.

To investigate the structural basis for the changes in the ARG content of *S. flexneri* 3a over time, we performed long-read (Nanopore) sequencing on three representative isolates based on their AMR gene profiles, year of isolation, and BAPS-group assignment. We then investigated similarity among contiguous sequences containing *mph*(A), which revealed that the *mph*(A) genes were located on plasmids. We found that the pKSR100-like plasmids from: the pKSR100 reference plasmid from *S. flexneri 3a*, and 2016 and 2020 UKHSA isolates of *S. flexneri* 3a were overall genetically similar (Fig. 4a). However, the 2016 and 2020 pKSR100-like plasmids did have some notable differences, particularly around the region carrying AMR genes (Fig. 4b). The pKSR100-like plasmid from the 2020 isolate was shorter (69,055 bp) than the 2016 version (73,462 bp) and had lost the *erm*(B) and *bla*_TEM-1_ genes, which were originally flanked by transposase genes. A *qacE* Δ-1-containing plasmid from a Global-BAPS-4 *S. flexneri* 3a isolate from 2020 was genetically distinct from the pKSR100 plasmids (Fig. 2), but contained a similar *mph*(*A*)-containing region also flanked by transposase genes. To explore the resolution more fully across the isolate collection (i.e., also in those isolates that were not long-read sequenced), we mapped isolates’ sequencing reads against pKSR100 which showed that the more recent isolates mapped against a lower proportion of pKSR100, likely reflecting the loss of *erm*(B) and *bla*_TEM-1_ captured in the individual isolate long-read comparisons (Supplementary Fig. 1), the decision to display percentage mapping of ≥60 was informed by class interval frequencies (Supplementary Fig. 1b) which allowed for a distinguishable colour gradient. This revealed that a newer, shorter plasmid with fewer AMR genes is now circulating in this MSM-associated *S. flexneri* 3a lineage. We visualised the changing plasmid content between MSMA-BAPS-1 and MSMA-BAPS-5 isolates (Supplementary Fig. 3).

## Discussion

We demonstrate distinct genomic subtypes of *S. flexneri* 3a associated with international travel, compared to MSM-associated shigellosis, in line with previous work^30^. This indicates that isolates associated with travel may be imported into the UK, which provides the basis for expansion of these initial travel-related clusters, in sexual transmission networks, hypothesised to create an environment of selection pressure from increased antimicrobial use for the treatment of other sexually transmissible infections, e.g., gonorrhoea^30,33^. Other factors such as the host immune response and gut microbiota also likely play a role.

The changes in the retention of ARGs between the pre- and post-2015 *S. flexneri* 3a isolates is intriguing. It appears that while the *erm*(B) and *bla*_TEM-1_ genes have been lost in the re-emergent isolates, *mph*(A) has been retained. We hypothesised that this could suggest that the *mph*(A) gene has been transferred from the plasmid pKSR100 into the core chromosomal genome of these *Shigella* isolates due to its importance to the survival of the bacterium, which was refuted by our long-read sequencing findings that revealed to the ongoing plasmid-borne nature of the resistance genes. However, our genomic analyses revealed that *mph*(A) was still being carried on plasmids. While the loss of *erm*(B) and *bla*_TEM-1_ could be purely stochastic, perhaps *erm*(B) and *bla*_TEM1_ have been lost due to being of lesser importance for bacterial survival. It is possible that azithromycin selection pressure has recently abated for sexually transmissible *Shigella* in the UK as treatment recommendations were recently updated for gonorrhoea. There are large active surveillance programmes for gonorrhoea that drove the emergence of azithromycin resistant *Shigella* as bystander resistance^30,34^. However, now monotherapy of 1 g of ceftriaxone is now recommended^35^, and ongoing bystander resistance is supported by the emergence of ESBL-producing strains^30^. Also, it could be that resistance to clinically relevant concentrations of their corresponding antibiotics is sufficiently mediated by other genes. For example, the *bla*_OXA-1_ chromosomal gene is highly conserved in Global-BAPS-1 *S. flexneri* 3a (Fig. 3), which confers resistance to a very similar substrate profile to that of *bla*_TEM-1_^36^, therefore, having both *bla*_OXA-1_ and *bla*_TEM-1_ is seemingly redundant, which would explain the loss of the latter, facilitated by its proximate transposases in the pKSR100-like plasmid. While the presence of both *erm*(B) and *mph*(A) is associated with a dual-action resulting in a higher MIC value for azithromycin^37^, it is clear that *mph*(A) alone can be sufficient in conferring a clinically resistant MIC value, which may explain the recent loss of *erm*(B) in *S. flexneri* 3a. The isolates remain susceptible to ciprofloxacin due to not having acquired relevant QRDR mutations, and are also susceptible to the third-generation cephalosporin ceftriaxone, having not acquired a gene in the *bla*_CTX-M_ family, unlike *S. sonnei* in the UK^23^, and internationally ^22^.

Although it is possible that stochastic population dynamics, travel-related importation, the AMR-related genetic composition changes, and non-AMR changes in the organisms (such as the fixation of the col156_1 plasmid in the Global-BAPS-1 group, and the chromosomal SNPs in MSMA-BAPS-5) may have driven the re-emergence of *S. flexneri 3a* in the UK. The amino acid substitution (alanine to threonine) in the lipopolysaccharide-associated outer membrane protein lptE may have an impact by generating some sort of altered LPS structure (and potentially immune escape). However, the BLOSUM score of ‘zero’ indicates that this substitution is likely conservative. It is also important to consider the potential role of waning host immunity in the dense sexual transmission networks facilitating endemic shigellosis transmission. Specifically, the hypothesis that waning immunity in the MSM population may permit re-emergence after a two year period. The recent MPox outbreak suggested that pathogens circulating in the dense MSM sexual transmission networks in the UK can quickly exhaust susceptible individuals in the population^38^, and in Israel, where *S. sonnei* is hyperendemic, short- to medium-term serotype specific immunity is thought to drive biennial peaks of *S. sonnei* infection^39^, consistent with the observations here for *S. flexneri* 3a. Shifting dominance of *S. flexneri* serotypes has also been described in MSM in Montréal^40^ and negative frequency-dependent selection (NFDS) may contribute to the re-emergence of rarer genotypes (as previously shown in *Streptococcus pneumoniae*)^41^. Further research involving mathematical modelling and immunological studies (the usefulness of these reviewed elsewhere^42^) are needed to elucidate the role of immunity and NFDS in the re-emergence of *S. flexneri* 3a and other serotypes of *S. flexneri* and species of *Shigella*.

It is difficult to identify a single dominating factor in the re-emergence of *S. flexneri* 3a in the UK. Our research investigated a variety of variables which may have contributed to this re-emergence: human population demographic changes, host immunity, and changes in the genetic makeup of the *S. flexneri* 3a isolates. We showed that the population demographic of individuals contracting *Shigella* spp. is similar to the previous outbreak. Though, the waning host immunity timelines, that have been demonstrated in previous work, may have contributed to this re-emergence. We see that there have been distinct genetic changes in the *S. flexneri* 3a, those being the acquisition and conservation of the col156_1 plasmid, SNPs which affect multiple genes via upstream and downstream locations, and a single substitution of an amino acid in the lipopolysaccharide protein lptE, and changes in the pKSR100 plasmid over time. Particularly, the loss of ARGs *erm*(B) and *bla*_TEM-1_, but retention of *mph*(A), which may confer a lower fitness cost to the bacteria and lead to its successful out-competition of other serotypes of *S. flexneri* and *Shigella* spp. We recommend that further research regarding the role of host immunity in the re-emergence of *Shigella* spp. is conducted through mathematical modelling and immunological studies to better predict and prevent future outbreaks of antimicrobial resistant, sexually transmissible enteric infections.

## Methods

### Data collection

Faecal samples of *S. flexneri* 3a were stored by the UK Health Security Agency (UKHSA) after being sent from hospitals and other diagnostic laboratories. Isolates with Sequence Read Run (SRR) numbers were sequenced by the UKHSA (2016–2020), while isolates with Experiment Run Archive (ERR) numbers were sequenced by the Wellcome Sanger Institute (2008–2013). The Second-Generation Surveillance System and Gastrointestinal Data Warehouse were used as sources of the metadata in this study. More severe disease is more likely to be reported, which may represent a bias in case numbers and hospitalisation rates. All isolate accession numbers are available in Supplementary Data Table 1.

### Data analysis

Where possible, samples from men who have sex with men (MSM) were identified from patient questionnaire data where sexual orientation was self-reported. Where this data was not available, samples from presumptive MSM were defined using previously validated parameters: males, aged 16 years and above, who had not declared any international travel in the last month^43^. Where travel history was not reported, this was assumed to mean that the patient did not travel.

### Illumina whole-genome sequencing

Whole-genome-sequencing of *Shigella* isolates became a part of the standard surveillance routine at the UKHSA from August 2015 onwards. Where samples of *S. flexneri* 3a are sent to the UKHSA from patients presenting with gastrointestinal illness, these samples had their DNA extracted for sequencing using an Illumina HiSeq 2500 machine. These Illumina reads were quality checked and trimmed and aligned to the *S. flexneri* serotype 2a strain 2457 T reference genome (GenBank: AE014073.1). These reads were uploaded to the NCBI website for download by other individuals.

### Nanopore sequencing

The two *S. flexneri* 3a isolates containing pKSR100-like plasmids and the one *qacE Δ-*1-containing plasmid underwent DNA extraction and Nanopore sequencing. Genomic DNA was extracted from overnight bacterial cultures using the MasterPure Complete DNA Purification Kit (Lucigen Corporations, Wisconsin, US) according to manufacturer’s instructions. DNA concentration, purity and quantity were assessed using Nanodrop spectrophotometry (DeNovix, Thermofisher, UK) and Qubit fluorometry (Invitrogen, US) according to manufacturer’s instructions. Sequencing libraries were prepared using the ligation sequencing kit - SQK-LSK109 - according to manufacturer’s instructions (Oxford Nanopore Technologies, Ltd, Oxford, UK). DNA libraries were sequenced using a MinION sequencer and FLO-MIN106 Flow Cell version R9.4.1 (Oxford Nanopore Technologies Ltd, Oxford, UK).

### Genomic analysis

Genomic analysis of the *S. flexneri* 3a isolates sequenced by Wellcome Sanger Institute (2008–2013), and the UKHSA (2016–2020), guided by data sharing agreements, was undertaken using a variety of methods, starting with downloading the FASTQ files available from the Sequence Read Archive (SRA) using the SRA toolkit (v 2.11.0) command: fastq-dump^44^. Trimmomatic (v 0.39)^45^ was used to ‘trim’ the sequences used, with the following parameters: 2:30:10 LEADING : 20 TRAILING : 20 SLIDINGWINDOW : 4 : 20 MINLEN : 40. Afterwards, the quality of the trimmed reads were then checked by using fastQC (v 0.11.9)^46^, then combined with multiQC (v 1.12)^47^. The reference genome used was of *S. flexneri* 3a, (GenBank: GCA_904066025.1). Mapping the experimental genomes with the reference genome was undertaken by using Burrows-Wheel Aligner (BWA, v 0.7.17)^48^, with PICARD (v 2.27.2)^49^ being used to remove PCR duplicates. Quality of the mapping was checked by using qualimap (v 2.2.2)^50^ bamqc.

### Phylogenetic tree creation

Variant calling was performed using samtools (v 1.11)^51^ and bcftools (v 1.9)^52^. SNP-sites -C (v. 2.5.1)^53^ was used on the core alignment multi-FASTA file to determine the invariant sites (IS). Using the parameters: *-fconst [IS1],[IS2],[IS3],[IS4] -keep-ident -bb 1000 -m GTR* + *F* + *I* + *G4*, the phylogenetic trees were created using IQtree (v 2.2.0.3)^54^ with the FASTA alignment output of filtered polymorphic sites generated by Gubbins (v 3.2.1)^55^. The genome of each isolate was assembled twice, once using UNICYCLER (v 0.5.0)^56^, and once using SPAdes (v. 3.13.0)^57^, where the quality of both sets of assembled genomes were then checked using QUAST (v 5.02)^58^. The UNICYCLER assemblies were used for all analyses except the PlasmidFinder/Abricate analyses, where the SPAdes assemblies were used. GraphSNP (v. 1.0)^59^ was used to extract average pairwise SNP distance data from the FASTA alignment output of filtered polymorphic sites.

### Gene detection

AMR, stress, and virulence genes were then identified using NCBI AMRFinder Plus (v 3.10.24)^60^, with the assembled genomes of each isolate and the following parameters: *--nucleotide assembly.fasta --organism Escherichia --plus*. Some (2.9%, *n* = 15/517) isolates were excluded from phylogenetic analysis due to quality control issues and not having an assigned SNP-address identifier. The SNP-address is a UKHSA in-house linkage-clustering system, previously described elsewhere^43^, and part of the UKHSA genomic surveillance pipeline^61^. The presence and absence of colicin genes in the genome assemblies was investigated - using a previously generated custom database^31^, embedded in Abricate (v. 1.0.1)^62^, using default parameters. The presence and absence of virulence genes was further investigated using VFDB^63^, embedded in Abricate (v.1.0.1)^62^.

### Antimicrobial susceptibility testing

To investigate whether genotypic resistance translated to phenotypic resistance, 20 isolates from varying AMR profiles were subject to antimicrobial susceptibility testing using MIC Test Strips (MTS) manufactured by Liofilchem®, completed to obtain MICs as outlined by manufacturer’s directions. Results were interpreted according to clinical breakpoint definitions (v. 12) provided by the European Committee on Antimicrobial Susceptibility Testing (EUCAST)^64^.

### Statistical analyses

Standard proportions were calculated. A 3 × 2 contingency table chi-square statistical test was performed upon the categorical data of the number of isolates with an MIC of >256 or <256, and their correlation with the presence of both *mph*(A) and *erm*(B), the presence of only *mph*(A), or the presence of neither. A *p* value of ≤0.05 was interpreted as being statistically significant.

### Assembly and annotation

The isolates with the plasmids of interest were assembled and annotated. The fast5 read files generated from the MinION instrument were base-called and demultiplexed with Guppy (v 5.0, Oxford Nanopore Technologies Ltd. Oxford, UK). Processed read files were filtered using Filtlong (v 0.2.0), then assembled using Flye (v 2.9)^65^. Racon (v 1.5.0)^66^ was used to polish contigs with the Nanopore reads. Medaka (v 1.6.1) was used to polish Racon polished contigs, with Nanopore reads specifying the model r941_min_sup_g507. Polypolish (v 0.5.0)^67^ was then used to polish with Illumina reads. The quality and statistics of each assembly were evaluated with QUAST (v 4.4.0)^58^ without a reference genome. Genomes were annotated using the Prokaryotic Genome Annotation Pipeline^68^. The complete genome sequence data have been submitted to the National Centre for Biotechnology (NCBI) and have been deposited at GenBank under the BioProject number PRJNA999503.

### Plasmid analyses

Plasmids (GBK files) were compared and visualised using Clinker^69^, embedded on the CompArative GEne Cluster Analysis Toolbox (CAGECAT, v 1.0) website, using default parameters. The 2020 pKSR100-like plasmid was assembled with Oxford Nanopore reads, and polished with Illumina reads from *S. flexneri* 3a (SRR12743036). The 2016 pKSR100-like plasmid was assembled with Oxford Nanopore reads, and polished with Illumina reads from *S. flexneri* 3a (SRR4897018). The 2014 pKSR100 reference plasmid was from *S. flexneri* 3a strain SF7955 (Plasmid accession LN624486.1). The 2020 *qacE Δ-1*-containing plasmid was assembled from Oxford Nanopore reads, and polished with Illumina reads from *S. flexneri* 3a (SRR1220418). PlasmidFinder database (v. 2.1.0)^70^, embedded in Abricate (v. 1.0.1)^62^, was used to determine the plasmid content of the genome assemblies, using default parameters. UNICYCLER (v. 0.5.0)^56^ plasmid assemblies were annotated using Prokka (v. 1.14.6)^71^, visualised singly in Artemis or as a group in Artemis Comparison Tool (ACT), (v. 18.2.0)^72^, gene annotations corroborated with the results from NCBI AMR Finder Plus (v 3.10.24)^60^, running protein amino acid sequences through BLASTp (online)^73^, and AlphaFold (online)^74^.

### SNP site and gene impact analyses

The SNPs between two clusters of isolates were identified first by combining the core genome alignment FASTA file output from Gubbins (v 3.2.1) with the TREEFILE output from IQTree (v 2.2.0.3) into TreeTime (v 0.11.3)^75^, using the ancestral sequence reconstruction function with default parameters. The resulting annotated tree .NEXUS file was visualised in interactive tree of life (iTOL, v 6.0)^76^ to identify the node ID labels of the ancestral nodes associated with each cluster of interest. The FASTA sequences of the corresponding nodes were extracted from the ancestral sequences .FASTA TreeTime (v 0.11.3) output. The ancestral node sequence associated with MSMA-BAPS-1 was annotated using Prokka (v. 1.14.6), while the ancestral node sequence associated with MSMA-BAPS-5 had synthetic Illumina reads generated using ART with the following parameters: art_illumina -p -i genome.fa -l 250 -f 100 -o output. -m 6000 -s 89. These synthetic reads were trimmed using Trimmomatic (v 0.39), and then mapped to the previously generated reference file using Burrows-Wheel Aligner (BWA, v 0.7.17)^48^, and PICARD (v 2.27.2), with variants being called using samtools (v 1.11)^51^ and bcftools (v 1.9)^52^. SNP identities and predicted effects of these variants were determined using SNPeff (v 5.10)^32^, with default parameters, where the annotated ancestral node of the original outbreak .GBK output of Prokka (v. 1.14.6) was used as a custom SNPeff reference database. The genes affected by these SNPs were identified by SNPeff (v 5.10)^32^, and paired with their corresponding encoded proteins by searching the gene name in UniProt^77^. The BLOcks SUbstitution Matrix 62 (BLOSUM)^78^ was used to evaluate the evolutionary frequency of amino acid substitutions present.

### Supplementary information

Supplementary Information

Supplementary Data 1

## Data Availability

The sequence data for each of the 517 samples used in this study can be downloaded by using the SRA-toolkit and running a FASTQ-dump on the accession numbers of each isolate used in this study. See Supplementary Data 1 for the ERR and SRR accession numbers of the isolates used in this work, including anonymised patient and sample metadata. The Oxford Nanopore derived assemblies of isolates used in comparative plasmid analyses can be found under BioProject accession PRJNA999503. Correspondence regarding isolates from the United Kingdom and the overall study should be directed to Dr. Claire Jenkins and Professor Kate S Baker.

## Code availability

No custom code was written to conduct this study. Details of bioinformatic packages and commands used can be found in the methods section of this article.
