# Supplementary material for: The re-emergence of sexually transmissible multidrug resistant Shigella flexneri 3a, England, United Kingdom
Source: NPJ Antimicrob Resist. 2024 Aug 2;2:20. doi: 10.1038/s44259-024-00038-3 (PMC11296952; doi:10.1038/s44259-024-00038-3)
Supplement: Supplementary file 1 — Supplementary Information [file 44259_2024_38_MOESM1_ESM.pdf]

Supplementary Information - The re-emergence of sexually transmissible, multidrug resistant *Shigella flexneri* 3a, England, United Kingdom

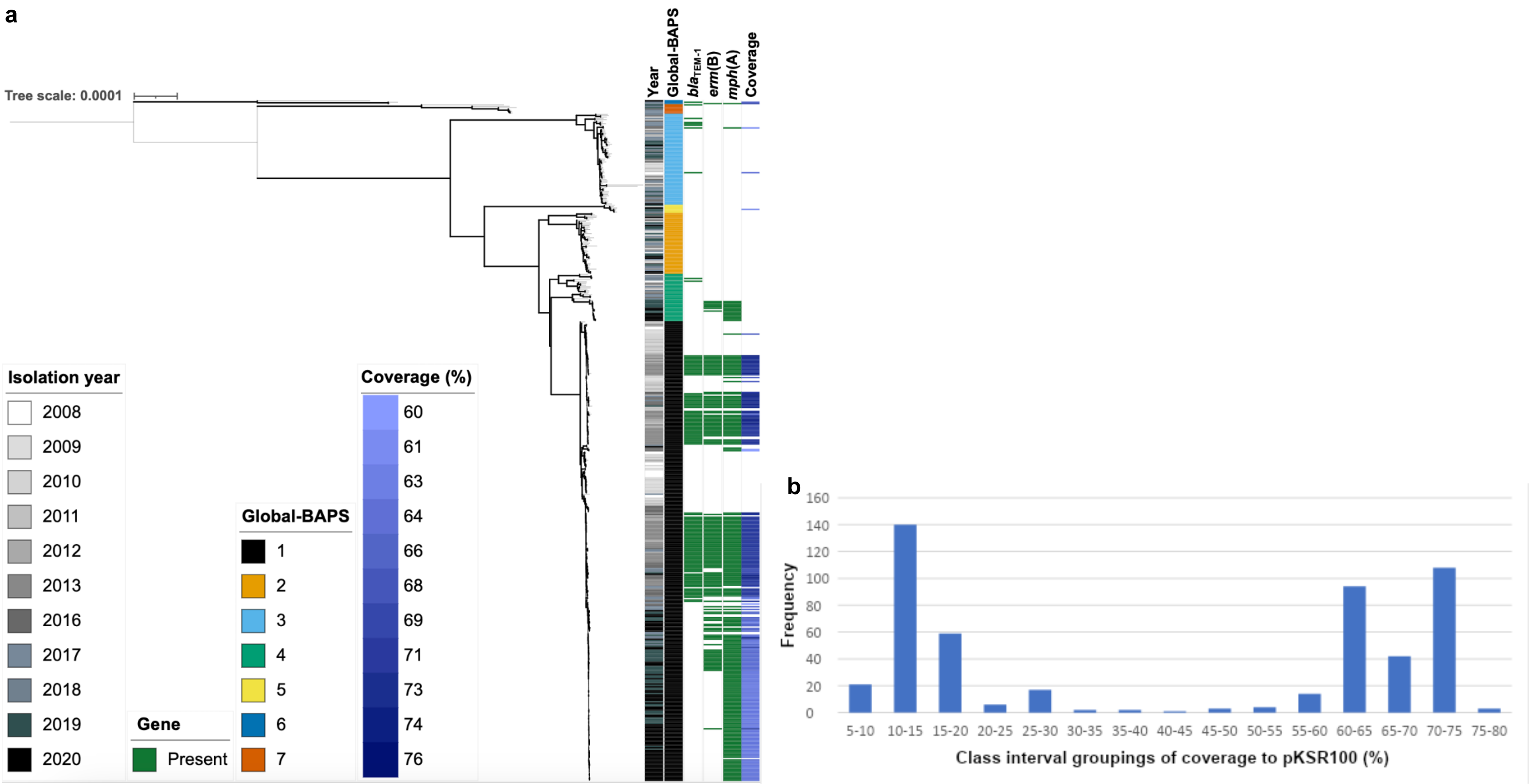

Supplementary Figure 1a. 1X coverage mapping of Illumina sequence reads of *S. flexneri* 3a isolates (*n* = 502) to a pKSR100 reference plasmid from *S. flexneri* 2a. Only coverage of ≥60% is displayed. Metadata tracks show year, BAPS subgroup, presence and absence of AMR genes associated with the pKSR100 plasmid, and the percentage mapping coverage at 1X depth of the *S. flexneri* 3a isolates to the pKSR100 reference plasmid from *S. flexneri* 2a. Emboldened branches represent a bootstrap value of ≥ 70 out of 100. Branches represent substitutions across a 22985 bp alignment. **Supplementary Figure 1b.** Histogram displaying the frequencies of varying groups of % coverage to pKSR100, including all isolates in the tree. Class interval groups of ≥ 60 were selected to be displayed on the phylogenetic tree.

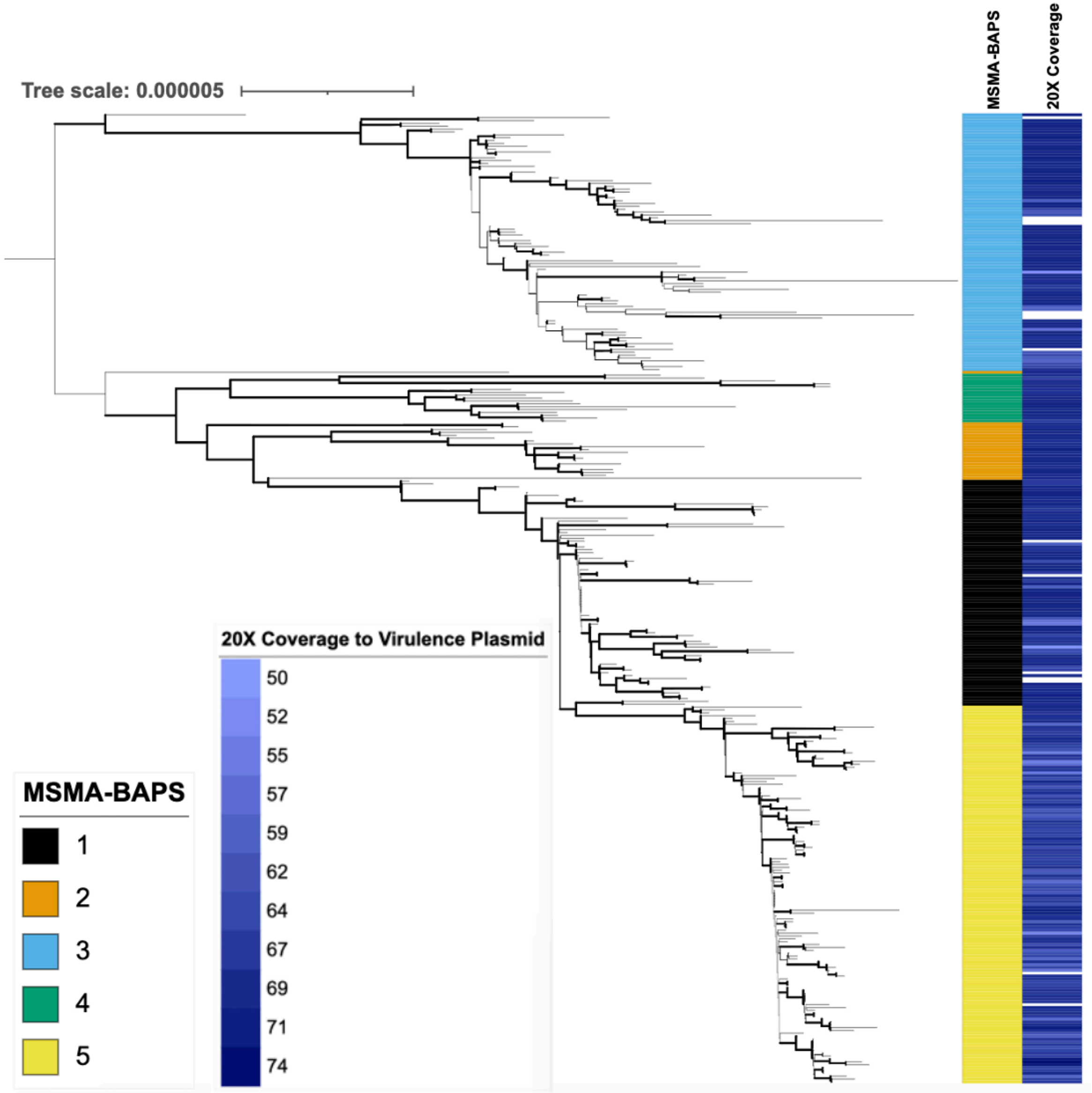

Supplementary Figure 2. 20X coverage mapping of Illumina sequence reads of *S. flexneri* 3a MSMA-BAPS isolates (*n* = 339) to a *S. flexneri* 3a virulence reference plasmid. Only coverage of ≥50% is displayed. Metadata tracks show MSMA-BAPS and the percentage mapping coverage at 20X depth of the *S. flexneri* 3a isolates to the *S. flexneri* 3a virulence reference plasmid. Emboldened branches represent a bootstrap value of ≥ 70 out of 100. Branches represent substitutions across a 22985 bp alignment. The reference genome used was 4522047bp in length.

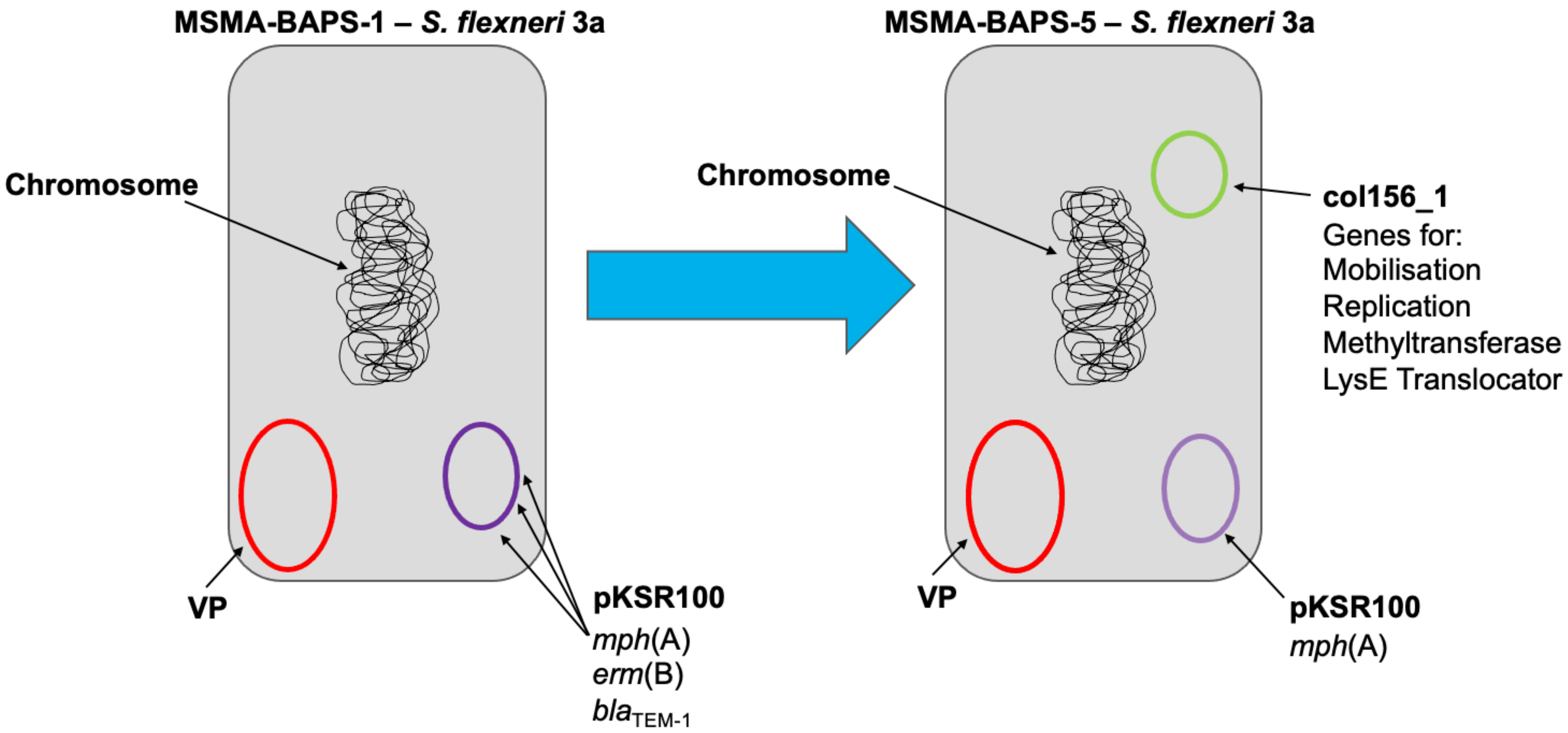

Supplementary Figure 3. A diagram outlining the plasmid-content similarities and differences between the average MSMA-BAPS-1 isolate and average MSMA-BAPS-5 isolate. Plasmids are visualised as: virulence plasmid (VP) in red, pKSR100 in purple, col156_1 in green and the core chromosome in black. Fading of pKSR100 represents the loss of AMR genes. Plasmid sizes are roughly approximated relative to their lengths.

Supplementary Table 1. SNP distance information within and between MSMA-BAPS-1 and MSMA-BAPS-5

	Intra-MSMA-BAPS-1	Intra-MSMA-BAPS-5	Inter-MSMA-BAPS-1 and MSMA-BAPS-5
**Average Pairwise Distance (SNPs)**	19	14	39
**Range (X - Y SNPs)**	0 - 63	0 - 41	20 - 76
**Interquartile Range (SNPs)**	19 (29 - 10)	11 (17 - 16)	13 (46 - 33)
**Median (SNPs)**	18	9	39

Supplementary Table 2. Changing proportions of MSMA-BAPS-1 and MSMA-BAPS-5 isolates carrying the col156_1 plasmid

MSMA-BAPS-Group	Total (n)	Presence of col156_1 (%)
1	79	5 (*n* = 4)
5	132	100 (*n* = 132)

Supplementary Table 3. Proportions of MSMA-BAPS-1 and MSMA-BAPS-5 isolates containing two highly conserved colicin genes

MSMA-BAPS-Group	Total (n)	Presence of tolR CDU39502.1 (%)	Presence of colicin I receptor EGI15668.1 (%)
1	79	100 (*n* = 79/79)	98.7 (*n* = 78/79)
5	132	100 (*n* = 132)	100 (*n* = 132)

Supplementary Table 4. Genes and their corresponding protein functions affected by two SNP variants identified in the ancestral node of MSMA-BAPS-5 in comparison to the ancestral node of MSMA-BAPS-1. Protein functions are as reported by UniProt. Impact levels are as reported by SNPeff.

Gene	Protein function	Location	Impact level
Hypothetical	Unknown	Upstream	Modifier
Hypothetical	Unknown	Upstream	Modifier
cobC	Adenosylcobalamin/alpha-ribazole phosphatase	Upstream	Modifier
degA	Amphoterin-induced protein 2	Upstream	Modifier
dsdA	D-serine dehydratase	Downstream	Modifier
dsdX	D-serine transporter DsdX	Downstream	Modifier
emrK	Probable multidrug resistance protein EmrK	Upstream	Modifier
emrY	Probable multidrug resistance protein EmrY	Upstream	Modifier
esiB_1	Secretory immunoglobulin A-binding protein EsiB	Downstream	Modifier
evgA	DNA-binding transcriptional activator EvgA	Upstream	Modifier
evgS	Sensor protein EvgS	Upstream	Modifier
holA	DNA polymerase III subunit delta	Upstream	Modifier
leuS	NAD(P)H-hydrate epimerase	Downstream	Modifier
lptE	LPS-assembly lipoprotein LptE	Missense	Moderate
mrdA	Peptidoglycan D,D-transpeptidase MrdA	Upstream	Modifier
nadD	Nicotinate-nucleotide adenylyltransferase	Upstream	Modifier
rlmH	Ribosomal RNA large subunit methyltransferase H	Upstream	Modifier
rsfS	Ribosomal silencing factor	Upstream	Modifier
yfdE_2	acetyl-CoA:oxalate CoA-transferase	Downstream	Modifier

Supplementary Table 5. Characteristics of the changed LPS-assembly lipoprotein LptE between MSMA-BAPS-1 and MSMA-BAPS-5.

**Gene**	*lptE*
**Protein**	LPS-assembly lipoprotein LptE
**SNP type**	Missense
**Amino acid change**	Alanine (A) to Threonine (T)
**BLOSUM score**	Zero
**Polarity change**	Non-polar to polar
**Hydropathy change**	Hydrophobic to hydrophilic
**Interaction with hydrogen change**	None, to hydro donor & acceptor

Supplementary Table 6. Antimicrobial susceptibility testing and genotypic AMR profiles of a subset of 20 representative isolates of the varying AMR profiles in Global-BAPS-1. Performed and reported according to European Committee on Antimicrobial Susceptibility Testing (EUCAST) guidance. All isolates were from males, aged 16-60, who had not travelled internationally in the last month. The antibiotics: Ciprofloxacin (CIP), Ceftriaxone (CRO), Azithromycin (AZM), Fosfomycin (FOS), Mecillinam (MEC), Ertapenem (ERT), Sulfamethoxazole-Trimethoprim (SXT) were used.

Metadata		Genes												Minimum Inhibitory Concentration (mg/L)						
**Accession**	**Year**	***bla*$_{OXA-1}$**	***catA1***	***aadA1***	***tet*(B)**	***bla*$_{TEM-1}$**	***erm*(B)**	***mph*(A)**	***dfrA17***	***sul1***	***aadA5***	***dfrA14***	***sul2***	**CIP**	**CRO**	**AZM**	**FOS**	**MEC**	**ERT**	**SXT**
ERR230553	2010	P	P	P	P	A	A	A	A	A	A	A	A	0.008	0.016	3	0.38	0.064	0.008	0.094
ERR230509	2008	P	P	P	P	A	A	A	A	A	A	A	A	0.008	0.016	3	0.5	0.125	0.008	0.094
ERR230486	2008	P	P	P	P	A	A	A	A	A	A	A	A	0.012	0.023	3	0.25	0.094	0.006	0.094
SRR7286393	2016	P	P	P	P	A	A	P	A	A	A	P	P	0.008	0.016	>256	0.5	0.064	0.006	>32
SRR4788220	2016	P	P	P	P	A	A	P	A	A	A	P	P	0.012	0.016	64	0.5	0.064	0.006	>32
ERR654275	2013	P	P	P	P	P	P	P	A	A	A	P	P	0.012	0.016	2	0.38	0.38	0.006	0.094
ERR654270	2013	P	P	P	P	P	P	P	A	A	A	A	A	0.016	0.032	>256	0.75	0.75	0.008	0.125
ERR654256	2013	A	A	P	P	P	P	P	P	P	P	A	P	0.008	0.016	>256	0.38	0.75	0.004	>32
ERR449050	2013	A	A	P	P	P	P	P	P	P	P	A	A	0.008	0.012	>256	0.5	0.75	0.003	>32
SRR9257780	2019	P	P	P	P	A	P	P	A	A	A	A	A	0.012	0.032	>256	0.75	0.064	0.008	0.094
SRR4897819	2016	P	P	P	P	P	P	P	A	A	A	A	A	0.008	0.016	>256	0.5	0.75	0.008	0.094
SRR4897018	2016	P	P	P	P	P	P	P	A	A	A	A	A	0.012	0.023	>256	0.75	0.75	0.006	0.094
SRR12743036	2020	P	P	P	P	A	A	P	A	A	A	A	A	0.012	0.023	32	0.5	0.064	0.008	0.094
SRR12683901	2020	P	P	P	P	A	A	P	A	A	A	A	A	0.008	0.023	24	1	0.125	0.008	>32
SRR12598696	2020	P	P	P	P	A	A	P	A	A	A	A	A	0.008	0.016	48	0.38	0.064	0.008	0.094
SRR12470239	2020	P	P	P	P	A	A	P	A	A	A	A	A	0.012	0.016	>256	0.5	0.094	0.006	0.094
SRR11832244	2020	P	P	P	P	A	A	P	A	A	A	A	A	0.012	0.023	32	0.38	0.094	0.008	0.094
SRR10858726	2019	P	P	P	P	A	A	P	A	A	A	A	A	0.008	0.023	32	0.5	0.064	0.008	0.094
SRR10419840	2019	P	P	P	P	A	P	P	A	A	A	A	A	0.012	0.032	>256	0.75	0.064	0.008	0.094
ERR449074	2013	P	P	P	P	P	P	P	A	A	A	A	A	0.008	0.016	>256	0.38	0.75	0.006	0.094

Absent
Present

Sensitive
Resistant
